# The Prevalence of ‘‘Food Addiction’’ during the COVID-19 Pandemic Measured Using the Yale Food Addiction Scale 2.0 (YFAS 2.0) among the Adult Population of Poland

**DOI:** 10.3390/nu13114115

**Published:** 2021-11-17

**Authors:** Magdalena Zielińska, Edyta Łuszczki, Anna Bartosiewicz, Justyna Wyszyńska, Katarzyna Dereń

**Affiliations:** Institute of Health Sciences, Medical College of Rzeszów University, 35-959 Rzeszów, Poland; eluszczki@ur.edu.pl (E.Ł.); abartosiewicz@ur.edu.pl (A.B.); jwyszynska@ur.edu.pl (J.W.); kderen@ur.edu.pl (K.D.)

**Keywords:** eating habits, eating behavior, psychology, COVID-19, food addiction, pandemic, overweight, obesity

## Abstract

The announcement of the coronavirus pandemic by the World Health Organization (WHO), ongoing restrictions and isolation led to a break with the daily routine, and suspension of social contacts, but also imposed new challenges on the population related to maintaining healthy eating habits. The purpose of the study was to assess the prevalence of “food addiction” (FA) during the COVID-19 pandemic in Poland in relation to several variables including depression. The method of analysis was a questionnaire containing original questions and the Yale Food Addiction Scale 2.0 (YFAS). A total of 1022 Polish residents aged 18–75 participated in the study (*N* = 1022; 93.7% women, 6.3% men). The prevalence of FA during the COVID-19 pandemic measured with the YFAS 2.0 scale was 14.1%. The average weight gain during the pandemic in 39% of respondents was 6.53 kg. Along with the increase in the value of the BMI index, the intensity of “food addiction” increased in the study group. People with depression had statistically significantly more FA symptoms than healthy people. This work may motivate future research to evaluate the association and potential overlap of “food addiction” and problem eating behaviors during the pandemic and the obesity problem.

## 1. Introduction

The COVID-19 disease caused by the SARS-CoV-2 virus has caused many unexpected changes around the world. The announcement of the coronavirus pandemic by the World Health Organization (WHO) on March 11, 2020, ongoing global restrictions and isolation led to a break with the daily routine, and the suspension of social contacts, but also imposed new challenges on the population related to maintaining healthy eating habits [1]. Quarantine and pandemic-related restrictions are key in limiting the spread of the virus, but at the same time they contribute to the deterioration of the quality of the diet, excessive food consumption and reduced physical activity [2,3,4]. Improper diet and sedentary lifestyle are closely related to both physical and mental health. The epidemiological situation in the world significantly influenced the behavior related to lifestyle, as well as the deterioration of mental health [5]. It has been found that during a pandemic, people with a history of mental health disorders tend to relapse [6,7,8,9,10,11,12]. Scientific evidence demonstrates a close correlation between unhealthy eating patterns, low-quality diets and the state of increased anxiety and emotional disorders [13]. Moreover, stress and sadness are associated with lower motivation to eat healthily and with a reduced feeling of pleasure while eating [3,14]. It has been shown that the pandemic situation is conducive to changes in consumers’ eating behavior, leading also to the accumulation of food supplies, and at the same time to changes conducive to weight gain [15,16,17]. It is well known that isolated and stressed people, as well as a significant proportion of the population during COVID-19 emergencies, often turn to substances or take “rewarding” actions to deal with negative feelings [17]. A number of factors related to the COVID-19 pandemic may contribute to the incidence of food addiction (FA). Although the concept of FA was introduced sixty-five years ago, its definition and implications are still under discussion, both in society and in the scientific community [18,19]. The term suggests that people may experience reactions (under the influence of excessive and uncontrolled consumption of extremely palatable food) similar to the addictive ones that are adequate to those seen with classic intoxicants, both at the behavioral and neurobiological levels [20,21,22,23,24,25,26,27,28]. The American Society for Addiction Medicine has defined addiction as “a curable, chronic medical disease involving the complex interactions between the brain, genetics, environment, and life experiences of an individual. Addicted people use substances or engage in behaviors that become compulsive and often persist despite harmful consequences” [29]. The symptoms of FA include not only overeating, but also intrusive thoughts about food, experiencing strong emotions related to eating, giving up interests, ignoring and neglecting other activities and relationships with the environment. The preoccupation with food becomes an important, integral part of many people’s lives, and other spheres of functioning are subordinated to it. Binge eating and a focus on eating are related to the simultaneous loss of control over this process, experienced by many people, especially in relation to the tasty products included in the YFAS 2.0 questionnaire, i.e., sweets, bread or other cereal products, salty snacks, fast food and sweetened drinks [30]. FA has been suggested to be associated with changes in the mesolimbic dopaminergic pathway that underpins the transition from reward-driven eating to impulsive and compulsive eating. The model is based on the assumption that a particular food category or certain food ingredient has a direct effect on the brain, introducing changes that ultimately take over behavior related to reward [28]. Some researchers argue that the main culprit is sugar, although the evidence of this theory is questionable and not entirely convincing [31,32,33]. Other studies show that the combination of sugars and fats in the Western diet increases the susceptibility to addictive eating behavior [34]. Foods high in both fat and carbohydrates have been shown to be more palatable than foods high in only fat or only carbohydrates, and foods containing a combination of these macronutrients had a greater effect on the activity of brain areas related to the reward center [34]. Studies have shown that highly processed, high-energy products with a high-glycemic load and high-fat content were most often associated with addiction-like eating behaviors, especially in people with elevated FA symptoms [35]. Moreover, staying indoors for long periods may increase the risk of compulsive overeating, high-calorie food consumption and thus the occurrence of FA [36,37,38]. The questions in the YFAS questionnaire were adjusted to assess the consumption of high-fat, high-carbohydrate foods and were analyzed by an expert panel. FA was assessed using the Yale Food Addiction Scale (YFAS). It was initially developed and validated in 2009 [26]. In 2016, YFAS 2.0 was developed to be consistent with the current addiction diagnostic criterion, described in the DSM-5 (Diagnostic and Statistical Manual of Mental Disorders) [39,40]. YFAS is the only available tool for measuring addictive eating behavior and is currently the best available measure for assessing “food addiction” [40]. YFAS has been translated and validated in European countries [41]. It is emphasized that FA is both an individual and collective problem that needs to be addressed at the social level [19]. The purpose of the study was to assess the prevalence of “food addiction” (FA) during the COVID-19 pandemic in Poland in relation to several variables including depression.

## 2. Materials and Methods

### 2.1. Study Participants and Exclusion Criteria

A total of 1066 residents from various regions of Poland took part in the study. After reading and providing their informed consent to participate in the study, participants completed an anonymous online survey. The link to the form (Google Forms online survey platform), preceded by a short description of the purpose of the study, was made available via social media (Facebook, Instagram) from 1 January 2021 to 20 June 2021 (i.e., after about 12 months of the pandemic). The participants could complete the questionnaire directly from their smartphone, tablet or computer. Ultimately, 1022 people were qualified for the study. Indeed, 44 participants who did not meet the study criteria were excluded. These were people under the age of 18, with eating disorders, as well as pregnant and breastfeeding women. The rejection criteria are argued for a physiological and psychological condition that may influence eating behavior and thus the identification of FA. 

### 2.2. Study Documentation

The personal data and data of the participants were anonymous in accordance with the General Data Protection Regulation of the European Parliament (GDPR 679/2016). The study was approved by the Bioethics Committee of the University of Rzeszów (Approval Code: Resolution No. 6/05/2021, Approval Date: 20 May 2021). Participants were allowed to withdraw at any stage of the study. 

### 2.3. Questionnaire and Principles for Determining the Degree of “Food Addiction”

The method of analysis was a questionnaire containing proprietary questions and the Yale Food Addiction Scale 2.0 (YFAS 2.0). The first part consisted of questions concerning gender, education, place of residence, lifestyle (e.g., changes in tobacco consumption, physical activity), professional situation related to the pandemic, anthropometric measurements and the presence of certain pathologies. The second part involved a 35-item Polish adaptation of the questionnaire Yale Food Addiction Scale Version 2.0 [42,43]. YFAS 2.0 takes into account the eating behavior of the last 12 months. Based on this part of the questionnaire, the degree of “food addiction” during the pandemic was determined. In YFAS 2.0, all 35 items are continuous with measures 0 to 7 (0—never, 1—less than monthly, 2—once per month, 3—two or three times per month, 4—once per week, 5—two to three times per week, 6—four to six times per week, 7—every day). The diagnostic result on this scale is within the range of 0–11: the higher the score, the higher the level of FA. The presence of no more than one symptom or the absence of a symptom 12 (taking the substance causes serious problems or stress) was classified as non-addiction, the presence of 2–3 symptoms and the occurrence of a symptom 12 was classified as mild addiction, the presence of 4–5 symptoms together with the symptom 12 addiction was classified as moderate and the occurrence of more symptoms with 12 was classified as severe addiction. 

### 2.4. Statistics Calculations

The calculations were made in the R ver. 3.6.0 statistical environment, with the PSPP program and MS Office 2019. *p* = 0.05 was adopted as the level of significance. Variables expressed at the ordinal or nominal level were analyzed using chi-square tests. Non-parametric tests (the Mann–Whitney U test or Kruskall–Wallis test) were used to analyze quantitative variables divided into groups. The selection of tests was made on the basis of the distribution of variables, which was verified with the Shapiro–Wilk test. 

## 3. Results

In total, 1022 inhabitants from various regions of Poland qualified for the study. The majority of the group were women (93.7%). A total of 94.2% of the respondents were people with secondary or higher education, 26.8% of the respondents were residents of rural areas and 32.9% of the group were residents of cities with more than 250,000 inhabitants. Based on the collected anthropometric data, the body mass index (BMI) was calculated using the formula: the quotient of body mass [kg] and height [m²]. The subjects were divided into groups according to the classification: underweight (BMI < 18.5 kg/m²), normal weight BMI (18.5–24.9 kg/m²), overweight BMI (25.0–29.9 kg/m^2^) and obesity (BMI ≥ 30.00 kg/m²). In total, 53.7% of the respondents were people with a normal body weight, 40% were overweight and obese and the smallest number of people were underweight (6.4%). The characteristics of the study group are described in Table 1.

Participants were 18–75 years of age (mean = 33.18 years, SD = 11.86 years). The average weight gain of people who gained weight during the pandemic (*N* = 399, 39% of respondents) was *M* = 6.53 kg (*Min* = 1 kg, *Max* = 30 kg, *SD* = 4.21 kg). Half of the people whose weight increased were characterized by addiction not lower than *Me* = 7.00, while half of the people whose weight did not change were characterized by addiction not higher than *Me* = 2.00. Half of those whose weight decreased were not more than *Me* = 3.00. There were significant statistical differences (*p* < 0.05) between people whose weight increased and the other subjects. Among people whose body weight increased during the pandemic, there were statistically significantly (*p* < 0.05) more symptoms of food addiction than among the other people. Table 2 presents the descriptive statistics for the study group of *N* = 1022 people, taking into account the average, minimum and maximum values, as well as the median values of variables such as age, weight, height, calculated BMI, weight gain during the pandemic and “food addiction”. 

The result was statistically significant (*p* = 0.001). People who were not diagnosed with FA accounted for 85.9% of the study group, people with severe addiction accounted for 12.9%, while people with moderate addiction 0.8% and mild addiction 0.4%. Presented below are the observed (real) and expected values, the result of the χ^2^ test for one sample and the residual graph (difference between the observed and expected values). Table 3 shows the distribution of the levels of “food addiction”.

A statistically significant correlation (*p* < 0.001) between the BMI index and “food addiction” was observed. The correlation was moderately strong, as evidenced by the value of the coefficient 0.3 ≤ | rho | ≤ 0.5. It was a positive correlation, which means that the increase in the value of BMI coincided with the increase in the severity of FA in the study group. The distribution of variables is presented in Table 4.

For the purposes of the study, three age categories were created: up to 25 years old, 26–35 years old and over 35 years old. There were statistically significant differences (*p* < 0.05) between people up to 25 years old and people aged 26–35 and over 35 years old. Half of the people up to 25 years of age were characterized by addiction not greater than *Me* = 3.00 (the other half of this age group by addiction not less than *Me* = 3.00), while half of the people in other age groups were characterized by addiction not less than *Me* = 4.00. Among people up to 25 years of age, there were statistically significantly (*p* < 0.05) fewer symptoms of “food addiction” than in other age groups. 

In terms of food addiction, the groups selected according to the place of residence differed statistically significantly (*p* < 0.05). A post hoc Bonferroni test was performed to determine exactly which groups contained significant differences. There were significant statistical differences (*p* < 0.05) between the inhabitants of cities with more than 250.000 people and inhabitants of villages and cities with up to 50 thousand. Half of the rural population was characterized by addiction not lower than *Me* = 5.00, while half of the rural population was characterized by addiction not greater than *Me* = 3.00, as well as half of the urban population up to 50.000. In people from cities over 250 thousand, there were statistically significantly (*p* < 0.05) more symptoms of food addiction than in people from rural areas and cities of up to 50 thousand people.

Half of people working or learning remotely were addicted to no more than *Me* = 3.00, while half of people who experienced deterioration of working conditions were addicted to at least *Me* = 5.50. People who lost their jobs, changed jobs, or had fewer jobs, had statistically significant (*p* < 0.05) more symptoms of FA than people working or learning in a remote system. There were statistically significant differences (*p* < 0.05) between people who worked or studied in a remote system and people who lost their job, had to change it, or had less work due to the pandemic. 

Half of the people who decreased their activity were characterized by addiction not less than *Me* = 5.00, while half of the people who did not change their activity were characterized by addiction not higher than *Me* = 2.00, and half of the people who increased their activity were not more than *Me* = 3.00. People who decreased physical activity during the pandemic had statistically significantly (*p* < 0.05) more symptoms of FA than other people. There were statistically significant differences (*p* < 0.05) between people who decreased physical activity during the pandemic and the other people. 

There was a statistically significant correlation (*p* < 0.05) between FA and weight gain. The correlation was moderately strong and positive. Thus, the more symptoms of addiction were noted in the studied subjects, the greater was their weight gain during the pandemic.

Half of the people with secondary or less than secondary education achieved a result not greater than Me = 3.00. The lowest result among this group was *Min* = 1.00, and the highest Max = 11.00. Half of the people with higher education obtained a result not lower than *Me* = 4.00. The lowest result was *Min* = 1.00 and the highest was *Max* = 11.00. The indicated differences, however, are not statistically significant (*p* > 0.05). This means that education does not statistically significantly differentiate the number of symptoms of food addiction. The distribution of the described variables is presented in the Table 5.

Half of the people who developed depression were characterized by addiction not lower than *Me* = 7.00. The lowest result among this group was *Min* = 1.00, and the highest *Max* = 11.00. Half of the people who did not develop depression had addiction not greater than *Me* = 3.00. The lowest result was *Min* = 1.00, and the highest was *Max* = 11.00. People with depression had statistically significantly (*p* < 0.05) more symptoms of FA than healthy people. The results are presented in Table 6.

## 4. Discussion

The results of the study are consistent with the current literature on the impact of the COVID-19 pandemic on emotional states and eating behavior. The existing scientific evidence shows that 43.5% of Polish respondents declared that they ate more during the quarantine, and 51.8% admitted to snacking more often between meals [44]. This study can illustrate the effects of this phenomenon, as it has been shown that the average weight gain during a pandemic in 39% of the respondents was 6.53 kg. Importantly, “food addiction” is defined by behavioral patterns and eating experiences, not body weight. However, some reports indicate that a much larger proportion of people meeting the FA criteria are obese [30]. Systematic reviews that included both clinical and population groups showed a mean prevalence of “food addiction” between 15 and 20%. In a meta-analysis of 36 studies, the mean incidence of FA diagnosis was 16.2% [45]. The amount of YFAS symptoms was correlated with body mass index (BMI) in non-clinical trials. It was variable among obese people or those with eating disorders [46]. In this study, an FA of 14.1% was recorded during the pandemic and it was shown that weight gain during the pandemic was associated with the occurrence of FA. With the increase in the BMI value, the severity of FA increased in the study group. In the general U.S. population, Schulte and Gearhardt found that 15% of people can have FA, regardless of BMI [46]. Pursey et al. determined the average incidence of FA in 19.9% of patients, moreover, the average incidence of FA was much more likely in overweight and obese people [47]. Kiyici et al. found that 32% of obese patients with an average BMI of 41.6 and seeking weight loss treatment had FA [48]. In a study by Hauck et al. the chances of reaching the YFAS 2.0 threshold for “food addiction” were higher in people not only obese but also underweight [21]. “Food addiction” is not the same as obesity, as FA can also affect people of normal weight; however, there seems to be an association between low and high BMI and the incidence of “food addiction”. In this study, FA has not been demonstrated in underweight people, presumably due to the exclusion of people with eating disorders (where BMI below normal may suggest the presence of, e.g., anorexia nervosa). Given that few people who are underweight were included in the current sample, it is difficult to draw conclusions about the potential link between “food addiction” and a low BMI. Moreover, little research has been done on FA among underweight people, thus providing only a small database for comparison purposes and increasing the need for future work. Studies have also found significant positive correlations between FA and depression or anxiety [45,49]. In the described study, people with depression (11.45% of respondents) had statistically significantly more symptoms of FA than healthy people. A systematic review of 62 studies from 17 countries, including China, Turkey, Iran, Spain and Italy, with a total of 162,639 participants, included reported rates of COVID-19 anxiety of 33% and depression of 28% [50]. A national study conducted in China (*N* = 52,730) revealed the incidence of mental stress at the level of 35% [51]. In a study by Berenson et al. women with FA more often had a higher level of depression compared to women who were not “addicted to food”, while no other features were significantly associated with FA [52]. The negative emotional effects of the global crisis caused by deaths, compulsory quarantine and economic disruption, along with feelings of isolation, fear of infection, stress and disrupted life, are likely to increase FA. Our results showed that stress related to COVID-19, i.e., job loss, was also associated with higher symptoms of FA. People who lost their jobs, changed jobs or had fewer jobs, had statistically significantly more symptoms of food addiction than people working or learning in a remote system. As with other reward stimuli, compulsive and uncontrolled overeating may reflect a dysfunctional strategy of coping with unpleasant states, experiencing uncertainty, despair, and/or self-regulation of emotions [53]. There is a significant positive relationship between “food addiction” and mental health symptoms. Moreover, there is extensive literature confirming that depression is associated with an increased risk of overeating during isolation [54,55]. Recent studies have confirmed the relationship between depressed mood and “pathological” eating habits, where depression was associated with an increased risk of overeating [56,57]. However, in a study by Rodríguez-Pérez et al. an improvement in eating behavior was observed during the COVID-19 pandemic [58]. Quarantine is associated with stress and depression, as well as an unhealthy diet and decreased physical activity. In the described study, almost half of the respondents (47.9%) declared a decrease in physical activity during the COVID-19 pandemic, while those who decreased their physical activity during the pandemic experienced statistically significantly more symptoms of FA than the remaining respondents. During the quarantine, there was a limited possibility of physical activity (closed gyms) and outdoor exercise, which resulted in limited physical activity. In a study by Li JTE et al. people with FA also reported significantly less physical activity [59]. Apart from influencing body weight and health aspects, physical activity may also play a role in the regulation of appetite, which may suggest a relationship with the occurrence of FA [60]. The pandemic negatively affected levels of physical activity, as confirmed by Lesser et al. in particular in outdoor activities that have been shown to have a protective effect on well-being [61]. Other researchers have also confirmed the negative impact of the epidemiological situation on mental health and the level of physical activity [62,63,64,65,66]. In addition to the above-mentioned factors, the study showed that in people from cities over 250,000 there were statistically significantly more FA symptoms during the COVID-19 pandemic than in people from rural areas and cities of up to 50,000. It can be assumed that this situation is related to the greater possibility of buying food in cities and ordering it at any time with delivery to a specific address. Additionally, it was shown that among people up to 25 years of age there were statistically significantly fewer symptoms of FA than in other age groups. Most publications focus on the diagnosis of FA among the youth or student population [67,68,69]. One study found that the rate of FA and the number of symptoms in women aged 18–34, 35–54 and over 55 years of age were significantly higher than in men in the same age groups, and there was no significant difference between the groups [70]. In a review by Pursey et al. the mean incidence of FA was lower in adults below 35 years of age compared to those above 35 years of age [47]. However, in one study, the frequency of FA diagnoses and the number of symptoms reported decreased with age: the diagnosis of FA was lower in the older age group [71]. The condition for exclusion from this study were people with eating disorders, because in many studies the diagnosis of FA among people diagnosed with a binge eating disorder (BED) is high and may reach 56.8% [72,73], while in the case of psychological bulimia it was 83.6% and 100% [74,75]. People with eating disorders could increase the incidence of FA in the analysis, which would distort the results focusing on factors related to the COVID-19 pandemic. One meta-analysis also showed that women were more often diagnosed with FA than men, which may be related to gender differences in hormonal profiles and/or eating patterns [47]. In a study by Penzenstadler et al. FA was diagnosed more often in women than in men [49]. The literature on eating disorders confirms that they are much more common in women than in men [76]. In a study by Yu et al. the gender differences in eating disorders among students were analyzed, emphasizing that female gender was a predictor of eating disorders and “food addiction” [77]. Men are less likely to recognize the symptoms associated with an eating disorder and are therefore less diagnosed and treated than women. The COVID-19 pandemic and the resulting quarantine restrictions may further exacerbate the endemic health crisis [78]. It should be noted that before the pandemic, insufficient levels of physical activity (low or inactivity and excessive screen time) and obesity were reported as a global public health problem [79,80]. Due to the problems described, the current COVID-19 pandemic and the occurrence of FA could further aggravate the situation and provoking another pandemic referred to as “Covibesity” by scientists, as “food addiction” is a likely causative factor in obesity [81,82]. Despite general concerns about the increasing prevalence and severity of addiction related to the COVID-19 risk, little data are available [83]. Our results seem to confirm general concerns about the negative impact of the COVID-19 crisis on addictive behavior, which suggests that this problem should be carefully monitored [22,36]. The main purpose of this type of research is to shed light on relevant phenomena in order to provide useful information on public health that can be taken into account by policymakers and health professionals when an emergency such as a pandemic occurs.

### Strengths and Limitations

The strength of our study is the relatively large sample of the respondents. Although our results are specific to the Polish population and should not be generalized to other populations, the obtained observations may be potentially useful in the design of research and interventions related to the pandemic and its consequences. To the best of our knowledge, this article is the first to investigate the prevalence of food addiction using YFAS 2.0 during a pandemic. However, there are also limitations to our research. Certain limitations are associated with potential errors and raise the credibility that may arise when data are self-reported by study participants in an online survey, such as self-reporting of height and weight. Additionally, in our study there was a large disproportion of the respondents in terms of gender, which made it impossible to determine the differences and relationships between sexes and FA and other factors. Although online surveys have many advantages (e.g., access to a large group of respondents from different parts of the country and to quarantined people, and it is a safe form of analysis during a pandemic), there are also disadvantages, such as selection bias, which should be taken into account [84]. For example, despite the increasing use and availability of the Internet throughout society, online questionnaires may be more accessible to some groups (e.g., young, middle-aged) than others (e.g., elderly) [85]. Similarly, and in line with the reported data, it has been shown that the percentage of responses to online surveys may be increased in favor of women, possibly due to gender differences in online behavior, e.g., women use social networks extensively, while men are more involved in online games [86,87,88,89]. Additionally, the online survey raises credibility concerns. More representative samples are needed to better understand the impact of a pandemic on the incidence of FA symptoms in the general population.

## 5. Conclusions

The diagnosis and symptoms of “food addiction” measured by the YFAS 2.0 method were significantly associated with increased BMI (overweight and obesity), middle and older age, low physical activity, job loss and depression. The results of the study highlight groups of people who may benefit from preventive and therapeutic measures related to FA. This work could motivate future research to assess the relationship and potential overlap between “food addiction” and problem eating behavior during the pandemic and the obesity problem. The results also show the necessity of nutritional education in order to cope with food shopping and proper nutrition in everyday life as well as in a crisis situation. When conducting nutrition education, it is necessary to encourage and motivate healthy lifestyle behaviors, especially to increase physical activity, even in a pandemic situation. 

## Figures and Tables

**Table 1:** Characteristics of the study group.

Body mass index (BMI)	Frequency	Percent
Underweight	65	6.40%
Normal weight	549	53.70%
Overweight	204	20.00%
Obesity	204	20.00%
The occupational situation during the pandemic	Frequency	Percent
No change	374	36.60%
Remote system	393	38.50%
Taking a job	4	0.40%
Change of a job	29	2.80%
Losing a job	81	7.90%
More work	2	0.20%
Less work	10	1.00%
A leave, e.g., childcare leave	17	1.70%
Non-working person	102	10.00%
Another	10	1.00%
Smoking	Frequency	Percent
Yes	134	13.10%
No	825	80.70%
I stopped smoking before the pandemic	43	4.20%
I started smoking during the pandemic	20	2.00%
Comorbidities	Frequency	Percent
Thyroid disease	187	18.30%
Type 2 diabetes	28	2.74%
Depression	117	11.45%
Hypercholesterolaemia	47	4.60%
Hypertension	85	8.32%
Elevated triglycerides	31	3.03%
Another	107	10.47%
Not applicable	617	60.37%
Sex	Frequency	Percent
Woman	958	93.70%
Man	64	6.30%
Education	Frequency	Percent
Primary education	6	0.60%
Lower secondary education	13	1.30%
Vocational education	41	4.00%
Secondary education	432	42.30%
Higher education	530	51.90%
Place of residence	Frequency	Percent
Village	274	26.80%
City up to 50,000	206	20.20%
City 50–100,000	99	9.70%
City 100–250,000	107	10.50%
City over 250,000	336	32.90%
Physical activity during a pandemic	Frequency	Percent
Increased	267	26.10%
Has not changed	265	25.90%
Decreased	490	47.90%
Change in body weight before the pandemic and now	Frequency	Percent
Increased	399	39.00%
Has not changed	378	37.00%
Decreased	245	24.00%

**Table 2:** Characteristics of the group with a description of the statistics, taking into account the average, minimum and maximum values as well as the values of the medians of the variables.

	*N*	*M*	*SD*	*Min*	*Maks*	*Me*
Age	1022	33.18	11.86	18.00	75.00	30.00
Body weight	1022	70.38	17.85	33.00	164.00	66.00
Height	1022	166.88	7.11	143.00	193.00	166.00
BMI	1022	25.20	5.90	12.27	54.17	23.74
Weight gain	399	6.53	4.21	1.00	30.00	5.00
“Food addiction”	1022	4.74	3.53	1.00	11.00	4.00

*N*—abundance; *M*—average; *SD*—standard deviation; *Min*—minimum; *Maks*—maximum; *Me*—median.

**Table 3:** Assessment of the occurrence of “food addiction”.

Variable Level	Values	*N*	Proportion	The Rest	Test Result
Lack	Observed	878.00	0.859	−622.50	*χ*^2^ = 2063.67 *df* = 3 *p* = 0.001
	Expected	255.50	0.250		
Mild	Observed	4.00	0.004	251.50	
	Expected	255.50	0.250		
Moderate	Observed	8.00	0.008	247.50	
	Expected	255.50	0.250		
Heavy	Observed	132.00	0.129	123.50	
	Expected	255.50	0.250		

*χ^2^*—test statistic; *df*—degrees of freedom; *N*—abundance; *p*—relevance.

**Table 4:** Correlation between BMI and FA in the study group.

		“Food Addiction”	
BMI	*rho*	0.351	***
	*p*	<0.001	

*rho*—Spearman’s correlation coefficient; *p*—relevance; *** *p* < 0.001.

**Table 5:** Correlation between “food addiction” and age range, place of residence, professional situation, physical activity and weight change.

		Descriptive Statistics					
	Age range	*χ* ^2^	*df*	*p*	*Min*	*Maks*	*Me*
“Food addiction”	up to 25 years	14.83	2	0.001	1.00	11.00	3.00
	26–35 years old				1.00	11.00	4.00
	over 35 years old				1.00	11.00	4.00
	Place of residence	*χ* ^2^	*df*	*p*	*Min*	*Maks*	*Me*
“Food addiction”	village	14.06	3	0.003	1.00	11.00	3.00
	city up to 50.000				1.00	11.00	3.00
	city 50–250 thousand				1.00	11.00	4.00
	a city with over 250.000				1.00	11.00	5.00
	Professional situation	*χ* ^2^	*df*	*p*	*Min*	*Maks*	*Me*
“Food addiction”	no change	17.18	3	0.001	1.00	11.00	4.00
	remote system				1.00	11.00	3.00
	loss/change/reduction				1.00	11.00	5.50
	the remaining				1.00	11.00	4.00
	Physical activity	*χ* ^2^	*df*	*p*	*Min*	*Maks*	*Me*
“Food addiction”	has increased	55.73	2	<0.001	1.00	11.00	3.00
	has not changed				1.00	11.00	2.00
	decreased				1.00	11.00	5.00
	Weight change	*χ* ^2^	*df*	*p*	*Min*	*Maks*	*Me*
“Food addiction”	has increased	152.96	2	<0.001	1.00	11.00	7.00
	has not changed				1.00	11.00	2.00
	decreased				1.00	11.00	3.00
	Education	*χ* ^2^	*df*	*p*	*Min*	*Maks*	*Me*
“Food addiction”	medium or lower	121,627.50		0.060	1.00	11.00	3.00
	higher				1.00	11.00	4.00

*χ*^2^—test statistic; *df*—degrees of freedom; *p*—statistical significance; *Min*—minimum score; *Maks*—maximum score; *Me*—median.

**Table 6:** Correlation between “food addiction” and the incidence of depression during a pandemic.

				Descriptive Statistics		
		*U*	*p*	*Min*	*Maks*	*Me*
Depression	“Food addiction”	36,488.50	<0.001			
	appeared			1.00	11.00	7.00
	did not occur			1.00	11.00	3.00

*U*—test statistic; *p*—statistical significance; *Me*—median; *Min*—minimum score; *Maks*—maximum score.

## Data Availability

The data presented in this study are available on request from the corresponding author. The data are not publicly available.

## References

[1] WHO Director (2020). Generals Opening Remarks at the Mission Briefing on COVID-19.

[2] Duerr H.P., Brockmann S.O., Piechotowski I., Schwehm M., Eichner M. (2007). Influenza pandemic intervention planning using InfluSim: Pharmaceutical and non- pharmaceutical interventions. BMC Infect. Dis..

[3] Robinson E., Boyland E., Chisholm A., Harrold J., Maloney N.G., Marty L., Mead B.R., Noonan R., Hardman C.A. (2021). Obesity, eating behavior and physical activity during COVID-19 lockdown: A study of UK adults. Appetite.

[4] Martínez-de-Quel Ó., Suárez-Iglesias D., López-Flores M., Pérez C.A. (2021). Physical activity, dietary habits and sleep quality before and during COVID-19 lockdown: A longitudinal study. Appetite.

[5] Flanagan. E.W., Beyl R.A., Fearnbach S.N., Altazan A.D., Martin C.K., Redman L.M. (2021). The Impact of COVID-19 Stay-At-Home Orders on Health Behaviors in Adults. Obesity.

[6] González-Sanguino C., Ausín B., Castellanos M.Á., Saiz J., López-Gómez A., Ugidos C., Muñoz M. (2020). Mental health consequences during the initial stage of the 2020 Coronavirus pandemic (COVID-19) in Spain. Brain Behav. Immun..

[7] Moccia L., Janiri D., Pepe M., Dattoli L., Molinaro M., De Martin V., Chieffo D., Janiri L., Fiorillo A., Sani G. (2020). Affective temperament, attachment style, and the psychological impact of the COVID-19 outbreak: An early report on the Italian general population. Brain Behav. Immun..

[8] Ozamiz-Etxebarria N., Dosil-Santamaria M., Picaza-Gorrochategui M., Idoiaga-Mondragon N. (2020). Stress, anxiety, and depression levels in the initial stage of the COVID-19 outbreak in a population sample in the northern Spain. Cad. Saude Publica.

[9] Rajkumar R.P. (2020). COVID-19 and mental health: A review of the existing literature. Asian J. Psychiatry.

[10] Temorshuizen J.D., Watson H.J., Thornton L.M., Borg S., Flatt R.E., MacDermod C.M., Bulik C.M. (2020). Early impact of COVID-19 on individuals with eating disorders: A survey of ~1000 individuals in the United States and The Netherlands. Int. J. Eat. Disord..

[11] Hao F., Tan W., Jiang L., Zhang L., Zhao X., Zou Y., Tam W. (2020). Do psychiatric patients experience more psychiatric symptoms during COVID-19 pandemic and lockdown? A case-control study with service and research implications for immunopsychiatry. Brain Behav. Immun..

[12] Shah K., Kamrai D., Mekala H., Mann B., Desai K., Patel R.S. (2020). Focus on mental health during the coronavirus (COVID-19) pandemic: Applying learnings from the past outbreaks. Cureus.

[13] Anton S.D., Miller P.M. (2005). Do negative emotions predict alcohol consumption, saturated fat intake, and physical activity in older adults?. Behav. Modif..

[14] Macht M. (2008). How emotions affect eating: A five-way model. Appetite.

[15] Naja F., Hamadeh R. (2020). Nutrition amid the COVID-19 pandemic: A multi-level framework for action. Eur. J. Clin. Nutr..

[16] Kohn S., Eaton J.L., Feroz S., Bainbridge A.A., Hoolachan J., Barnett D.J. (2012). Personal disaster preparedness: An integrative review of the literature. Disaster Med. Public Health Prep..

[17] Đogaš Z., Lušić Kalcina L., Pavlinac Dodig I., Demirović S., Madirazza K., Valić M., Pecotić R. (2020). The effect of COVID-19 lockdown on lifestyle and mood in Croatian general population: A cross-sectional study. Croat. Med. J..

[18] Randolph T.G. (1956). The descriptive features of food addiction; addictive eating and drinking. Q. J. Stud. Alcohol.

[19] Gordon E.L., Ariel-Donges A.H., Bauman V., Merlo L.J. (2018). What Is the Evidence for “Food Addiction?” A Systematic Review. Nutrients.

[20] Piccinni A., Bucchi R., Fini C., Vanelli F., Mauri M., Stallone T., Cavallo E.D., Claudio C. (2021). Food addiction and psychiatric comorbidities: A review of current evidence. Eat. Weight. Disord..

[21] Hauck C., Cook B., Ellrott T. (2020). Food addiction, eating addiction and eating disorders. Proc. Nutr. Soc..

[22] Volkow N.D., Wang G.J., Tomasi D., Baler R.D. (2013). Obesity and addiction: Neurobiological overlaps. Obes. Rev..

[23] Pedram P., Wadden D., Amini P., Gulliver W., Randell E., Cahill F., Vasdev S., Goodridge A., Carter J.C., Zhai G. (2013). Food addiction: Its prevalence and significant association with obesity in the general population. PLoS ONE.

[24] Avena N.M. (2011). Food and addiction: Implications and relevance to eating disorders and obesity. Curr. Drug Abus. Rev..

[25] Davis C., Curtis C., Levitan R.D., Carter J.C., Kaplan A.S., Kennedy J.L. (2011). Evidence that ‘food addiction’ is a valid phenotype of obesity. Appetite.

[26] Gearhardt A.N., Corbin W.R., Brownell K.D. (2009). Food addiction: An examination of the diagnostic criteria for dependence. J. Addict. Med..

[27] Blumenthal D.M., Gold M.S. (2010). Neurobiology of food addiction. Curr. Opin. Clin. Nutr. Metab. Care.

[28] Fortuna J.L. (2012). The obesity epidemic and food addiction: Clinical similarities to drug dependence. J. Psychoact. Drugs.

[29] American Society of Addition Medicine Definition of Addiction. https://www.asam.org/quality-practice/definition-of-addiction.

[30] Davis C. (2014). Evolutionary and neuropsychological perspectives on addictive behaviors and addictive substances: Relevance to the “food addiction” construct. Subst. Abus. Rehabil..

[31] Ahmed S.H., Guillem K., Vandaele Y. (2013). Sugar addiction: Pushing the drug-sugar analogy to the limit. Curr. Opin. Clin. Nutr. Metab. Care.

[32] Colantuoni C., Rada P., McCarthy J., Patten C., Avena N.M., Chadeayne A., Hoebel B.G. (2002). Evidence that intermittent, excessive sugar intake causes endogenous opioid dependence. Obes. Res..

[33] Westwater M.L., Fletcher P.C., Ziauddeen H. (2016). Sugar addiction: The state of the science. Eur. J. Nutr..

[34] Di Feliceantonio A.G., Coppin G., Rigoux L., Thanarajah S.E., Dagher A., Tittgemeyer M., Small D.M. (2018). Supra-additive effects of combining fat and carbohydrate on food reward. Cell Metab..

[35] Schulte E.M., Avena N.M., Gearhardt A.N. (2015). Which foods may be addictive? The roles of processing, fat content, and glycemic load. PLoS ONE.

[36] Lippi G., Henry B.M., Bovo C., Sanchis-Gomar F. (2020). Health risks and potential remedies during prolonged lockdowns for coronavirus disease 2019 (COVID-19). Diagnosis.

[37] Pearl R.L. (2020). Weight Stigma and the “Quarantine-15”. Obesity.

[38] Gordon E.L., Lent M.R., Merlo L.J. (2020). The Effect of Food Composition and Behavior on Neurobiological Response to Food: A Review of Recent Research. Curr. Nutr. Rep..

[39] Meule A., Heckel D., Kubler A. (2012). Factor structure and item analysis of the Yale Food Addiction Scale in obese candidates for bariatric surgery. Eur. Eat. Disord. Rev..

[40] Gearhardt A.N., Corbin W.R., Brownell K.D. (2016). Development of the yale food addiction scale version 2.0. Psychol Addict. Behav..

[41] Gearhardt A.N., Corbin W.R., Brownell K.D. (2009). Preliminary validation of the Yale food addiction scale. Appetite.

[42] Buczny J., Matyjanka C., Baranowska C. (2017). Polska adaptacja Full Yale Food Addiction Scale Version (YFAS) 2.0. SWPS Uniwersytet Humanistycznospołeczny, Wydział Zamiejscowy w Sopocie. https://www.researchgate.net/publication/324890291_Polska_adaptacja_Full_Yale_Food_Addiction_Scale_Version_YFAS_20.

[43] Poprawa R.W., Lewandowska B., Włodarczyk M., Tutka K. (2020). A polish adaptation and validation of the Yale Food Addiction Scale 2.0, Institute of Psychology. Alcohol Drug Addict..

[44] Sidor A., Rzymski P. (2020). Dietary Choices and Habits during COVID-19 Lockdown: Experience from Poland. Nutrients.

[45] Burrows T., Kay-Lambkin F., Pursey K., Skinner J., Dayas C. (2018). Food addiction and associations with mental health symptoms: A systematic review with meta-analysis. J. Hum. Nutr. Diet. Off. J. Br. Diet. Assoc..

[46] Schulte E.M., Gearhardt A.N. (2018). Associations of Food Addiction in a Sample Recruited to Be Nationally Representative of the United States. Eur. Eat. Disord. Rev. J. Eat. Disord. Assoc..

[47] Pursey K.M., Stanwell P., Gearhardt A.N., Collins C.E., Burrows T.L. (2014). The prevalence of food addiction as assessed by the Yale Food Addiction Scale: A systematic review. Nutrients.

[48] Kiyici S., Koca N., Sigirli D., Aslan B.B., Guclu M., Kisakol G. (2020). Food Addiction Correlates with Psychosocial Functioning More Than Metabolic Parameters in Patients with Obesity. Metab. Syndr. Relat. Disord..

[49] Penzenstadler L., Soares C., Karila L., Khazaal Y. (2019). Systematic Review of Food Addiction as Measured with the Yale Food Addiction Scale: Implications for the Food Addiction Construct. Curr. Neuropharmacol..

[50] Luo M., Guo L., Yu M., Jiang W., Wang H. (2020). The psychological and mental impact of coronavirus disease 2019 (COVID-19) on medical staff and general public—A systematic review and meta-analysis. Psychiatry Res..

[51] Qiu J., Shen B., Zhao M., Wang Z., Xie B., Xu Y. (2020). A nationwide survey of psychological distress among Chinese people in the COVID-19 epidemic: Implications and policy recommendations. Gen. Psychiatry.

[52] Berenson A.B., Laz T.H., Pohlmeier A.M., Rahman M., Cunningham K.A. (2015). Prevalence of Food Addiction among Low-Income Reproductive-Aged Women. J. Womens Health.

[53] Imperatori C., Fabbricatore M., Vumbaca V., Innamorati M., Contardi A., Farina B. (2016). Food Addiction: Definition, measurement and prevalence in healthy subjects and in patients with eating disorders. Riv. Psichiatr..

[54] Baenas I., Caravaca-Sanz E., Granero R., Sánchez I., Riesco N., Testa G., Vintró-Alcaraz C., Treasure J., Jiménez-Murcia S., Fernández-Aranda F. (2020). COVID-19 and eating disorders during confinement: Analysis of factors associated with resilience and aggravation of symptoms. Eur. Eat. Disord. Rev..

[55] Mengin A., Allé M.C., Rolling J., Ligier F., Schroder C., Lalanne L., Berna F., Jardri R., Vaiva G., Geoffroy P.A. (2020). Conséquences psychopathologiques du confinement [Psychopathological consequences of confinement]. Encephale.

[56] De Pasquale C., Sciacca F., Conti D., Pistorio M.L., Hichy Z., Cardullo R.L., Di Nuovo S. (2021). Relations Between Mood States and Eating Behavior During COVID-19 Pandemic in a Sample of Italian College Students. Front. Psychol..

[57] Mills J.G., Thomas S.J., Larkin T.A., Deng C. (2020). Overeating and food addiction in Major Depressive Disorder: Links to peripheral dopamine. Appetite.

[58] Rodríguez-Pérez C., Molina-Montes E., Verardo V., Artacho R., García-Villanova B., Guerra-Hernández E.J., Ruíz-López M.D. (2020). Changes in Dietary Behaviours during the COVID-19 Outbreak Confinement in the Spanish COVIDiet Study. Nutrients.

[59] Li J.T.E., Pursey K.M., Duncan M.J., Burrows T. (2018). Addictive Eating and Its Relation to Physical Activity and Sleep Behavior. Nutrients.

[60] Wiklund P. (2016). The role of physical activity and exercise in obesity and weight management: Time for critical appraisal. J. Sport Health Sci..

[61] Lesser I.A., Nienhuis C.P. (2020). The Impact of COVID-19 on Physical Activity Behavior and Well-Being of Canadians. Int. J. Environ. Res. Public Health.

[62] Ravalli S., Musumeci G. (2020). Coronavirus Outbreak in Italy: Physiological Benefits of Home-Based Exercise during Pandemic. J. Funct. Morphol. Kinesiol..

[63] Duncan G.E., Avery A.R., Seto E., Tsang S. (2020). Perceived change in physical activity levels and mental health during COVID-19: Findings among adult twin pairs. PLoS ONE.

[64] López-Bueno R., Calatayud J., Ezzatvar Y., Casajús J.A., Smith L., Andersen L.L., López-Sánchez G.F. (2020). Association Between Current Physical Activity and Current Perceived Anxiety and Mood in the Initial Phase of COVID-19 Confinement. Front. Psychiatry.

[65] Pieh C., Budimir S., Probst T. (2020). The effect of age, gender, income, work, and physical activity on mental health during coronavirus disease (COVID-19) lockdown in Austria. J. Psychosom. Res..

[66] Ammar A., Brach M., Trabelsi K., Chtourou H., Boukhris O., Masmoudi L., Bouaziz B., Bentlage E., How D., Ahmed M. (2020). Effects of COVID-19 Home Confinement on Eating Behaviour and Physical Activity: Results of the ECLB-COVID19 International Online Survey. Nutrients.

[67] Borisenkov M.F., Popov S.V., Pecherkina A.A., Dorogina O.I., Martinson E.A., Vetosheva V.I., Gubin D.G., Solovieva S.V., Turovinina E.F., Symaniuk E.E. (2020). Food addiction in young adult residents of Russia: Associations with emotional and anthropometric characteristics. Eur. Eat. Disord. Rev..

[68] Bourdier L., Orri M., Carre A., Gearhardt A.N., Romo L., Dantzer C., Berthoz S. (2018). Are emotionally driven and addictive-like eating behaviors the missing links between psychological distress and greater body weight?. Appetite.

[69] Lin Y.S., Tung Y.T., Yen Y.C., Chien Y.W. (2020). Food Addiction Mediates the Relationship between Perceived Stress and Body Mass Index in Taiwan Young Adults. Nutrients.

[70] Sanlier N., Turkozu D., Toka O. (2016). Body image, food addiction, depression, and body mass index in university students. Ecol. Food Nutr..

[71] Flint A.J., Gearhardt A., Corbin W., Brownell K., Field A., Rimm E. (2014). Food addiction scale measurement in two cohorts of middleaged and older women. Am. J. Clin. Nutr..

[72] Gearhardt A.N., White M.A., Masheb R.M., Morgan P.T., Crosby R.D., Grilo C.M. (2012). An examination of the food addiction construct in obese patients with binge eating disorder. Int. J. Eat. Disord..

[73] Gearhardt A.N., White M.A., Masheb R.M., Grilo C.M. (2013). An examination of food addiction in a racially diverse sample of obese patients with binge eating disorder in primary care settings. Compr. Psychiatry.

[74] Gearhardt A.N., Boswell R.G., White M.A. (2014). The association of “food addiction” with disordered eating and body mass index. Eat. Behav..

[75] Meule A., von Rezori V., Blechert J. (2014). Food addiction and bulimia nervosa. Eur. Eat. Disord. Rev..

[76] Striegel-Moore R.H., Rosselli F., Perrin N., DeBar L., Wilson G.T., May A., Kraemer H.C. (2009). Gender difference in the prevalence of eating disorder symptoms. Int. J. Eat. Disord..

[77] Yu Z., Indelicato N.A., Fuglestad P., Tan M., Bane L., Stice C. (2018). Sex differences in disordered eating and food addiction among college students. Appetite.

[78] Brooks S.K., Webster R.K., Smith L.E., Woodland L., Wessely S., Greenberg N., Rubin J.R. (2020). The psychological impact of quarantine and how to reduce it: Rapid review of the evidence. Lancet.

[79] Guthold R., Stevens G.A., Riley L.M., Bull F.C. (2018). Worldwide trends in insufficient physical activity from 2001 to 2016: A pooled analysis of 358 population-based surveys with 1·9 million participants. Lancet Glob. Health.

[80] Finkelstein E.A., Khavjou O.A., Thompson H., Trogdon J.G., Pan L., Sherry B., Dietz W. (2012). Obesity and severe obesity forecasts through 2030. Am. J. Prev. Med..

[81] Khan M.A., Moverley Smith J.E. (2020). “Covibesity”, a new pandemic. Obes. Med..

[82] Lennerz B., Lennerz J.K. (2018). Food Addiction, High-Glycemic-Index Carbohydrates, and Obesity. Clin. Chem..

[83] Marsden J., Darke S., Hall W., Hickman M., Holmes J., Humphreys K., Neale J., Tucker J., West R. (2020). Mitigating and learning from the impact of COVID-19 infection on addictive disorders. Addiction.

[84] Wright K.B. (2005). Researching Internet-based populations: Advantages and disadvantages of online survey research, online questionnaire authoring software packages, and web survey services. J. Comput. Mediat. Commun..

[85] Remillard M.L., Mazor K.M., Cutrona S.L., Gurwitz J.H., Tjia J. (2014). Systematic review of the use of online questionnaires of older adults. J. Am. Geriatr. Soc..

[86] Van Mol C. (2017). Improving web survey efficiency: The impact of an extra reminder and reminder content on web survey response. Int. J. Sci. Res..

[87] Muscanell N.L., Guadagno R.E. (2012). Make new friends or keep the old: Gender and personality differences in social networking use. Comput. Hum. Behav..

[88] Kimbrough A.M., Guadagno R.E., Muscanell N.L., Dill J. (2013). Gender differences in mediated communication: Women connect more than do men. Comput. Hum. Behav..

[89] Dufour M., Brunelle N., Tremblay J., Leclerc D., Cousineau M.M., Khazaal Y., Légaré A.A., Rousseau M., Berbiche D. (2016). Gender Difference in Internet Use and Internet Problems among Quebec High School Students. Can. J. Psychiatry.

